# Learning in Adverse Circumstances: Impaired by Learning With Anxiety, Maladaptive Cognitions, and Emotions, but Supported by Self-Concept and Motivation

**DOI:** 10.3389/fpsyg.2022.850578

**Published:** 2022-04-14

**Authors:** Manuela Paechter, Hellen Phan-Lesti, Bernhard Ertl, Daniel Macher, Smirna Malkoc, Ilona Papousek

**Affiliations:** ^1^Educational Psychology Unit, Department of Psychology, University of Graz, Graz, Austria; ^2^Department of Education, Ludwig Maximilian University of Munich, Munich, Germany; ^3^Department of Education, Universität der Bundeswehr München, Neubiberg, Germany; ^4^Institute for Practical Education and Action Research, University College of Teacher Education Styria, Graz, Austria; ^5^Biological Psychology Unit, Department of Psychology, University of Graz, Graz, Austria

**Keywords:** academic self-concept, motivation, proneness to anxiety, intrusion and hyperarousal, achievement emotions, academic performance

## Abstract

The COVID-19 summer semester 2020 posed many challenges and uncertainties, quite unexpectedly and suddenly. In a sample of 314 psychology students, it was investigated how they experienced learning and preparing for an end-of-semester exam, which emotions and strain they experienced, how academic performance was affected, and how personal antecedents of learning as important facets of a learner’s identity could support or prevent overcoming adverse circumstances of learning. The participants of the study filled in a questionnaire about their achievement emotions and strain they experienced during learning and exam preparation as well as academic self-concept, motivation, gender, proneness to anxiety. Points achieved in the exam were also recorded. The interaction between the variables was investigated by a structural equation model. It showed that the investigated variables can be distinguished into two groups, variables that contribute mainly negatively to performance and variables with a positive contribution. Strain experienced during the COVID-19 pandemic and unpleasant emotions “belong together” in the sense that they inhibit academic performance directly or indirectly. Proneness to anxiety in academic situations was related with higher levels of mental, emotional, and physical disturbances due to the COVID-19 situation. In contrast, motivation and a high academic self-concept acted as support for learning and performance. Both contribute to pleasant achievement emotions in the learning situation; moreover, motivation had a direct relationship to academic performance. The results from the present study do not only provide insight into important students’ personal dispositions and their role for learning in adverse circumstances but also give advice how to strengthen students for successful learning.

## Introduction

In Austria, the summer semester 2020 had just begun when on 11 March, COVID-19 was declared a global pandemic by the World Health Organization (World Health Organization [WHO], 2020). To slow the spread of the virus, restrictions on many everyday activities were announced for all citizens by the Austrian federal government. In connection with these developments, all universities closed their campuses and switched to distance learning within the span of about a single day (Feldhammer-Kahr et al., 2021). With closures of academic institutions and wider social restrictions in place, students experienced difficulties and insecurities concerning their studies, ranging from whether courses would still be continued or examinations be held to how to cope with the (until then rather unfamiliar) online-learning conditions. Students were severely affected by the outbreak of the pandemic in their academic and social life and were exposed to a large strain on their wellbeing (Vanaken et al., 2020; Chahal et al., 2021; Nia et al., 2021). Just as the population in general, university students were affected by affective sequelae of the circumstances during the pandemic, such as excessive feelings of stress or anxiety. Exacerbation of anxiety in the wake of the pandemic was related to usage of maladaptive cognition and coping strategies (Dinu et al., 2021; Godoy et al., 2021; Holt-Gosselin et al., 2021). These hitherto unknown circumstances of learning posed a threat to wellbeing and mental health which subsequently may have also impacted students’ learning and academic achievement (Andrews and Wilding, 2004; Barch et al., 2019; Charbonnier et al., 2022).

With this background, the present study investigates in a sample of psychology students how they experienced learning and preparing for an exam at the end of the summer semester 2020, which emotions they experienced, to which degree they felt strain and impairments, and how academic performance was affected. A focus was on the question to which degree antecedents of learning like proneness to anxiety, academic self-concept, or motivation may support or hinder learning in adverse circumstances.

## Learning and Performance in Adverse Circumstances

### Hyperarousal and Intrusion as Reactions to Strain

Drastic events like the COVID-19 pandemic influence an individual’s cognitive, emotional, and physiological processes in various ways. Already former pandemics like the H1N1 influenza or the acute respiratory syndrome (SARS) epidemic in 2003 had the potential to influence not only physical health but also mental health of the general population (Lau et al., 2010; Vanaken et al., 2020; Charbonnier et al., 2022). The few studies on the impact of the COVID-19 pandemic show a variety of strain-related responses in the population of different countries (e.g., for Belgium, Vanaken et al., 2020; China, Wang et al., 2019; Iran, Nia et al., 2021). Typical response patterns to such disturbances and strain manifest themselves in different ways; for example, as intrusion, i.e., intrusive thoughts, a preoccupation with the traumatic events, unwanted thinking about them, disrupting experiences like flashbacks to the difficult situations, frequent thoughts and images about the experience, related feelings about the experience, and/or dreaming about the events (Horowitz et al., 1979; Nia et al., 2021). Such intrusive disturbances may be accompanied by hyperarousal, i.e., troubles in concentrating, feeling jumpy, irritable, and angry, or physical reactions (Nia et al., 2021) as well as by attempts to avoid thoughts about the current situation, to remove the events from memory, or denying the meaning and consequences of the stressful events. Intrusion and hyperarousal concern cognition, emotion, as well as physiological processes (Sundin and Horowitz, 2003).

### Emotions in Learning Processes

Increased levels of distress and strain may interfere with students’ assessments of a learning situation, with their learning behaviors, their emotions and learning outcomes. One speaks of achievement emotions and of proximal learning factors when describing emotions experienced in learning-related situations (Pekrun et al., 2007; Kamtsios and Karagiannopoulou, 2020; Rentzios and Karagiannopoulou, 2021). Achievement emotions can be distinguished into pleasant or unpleasant ones (Putwain et al., 2021).

Examples for unpleasant achievement emotions are anxiety, anger, shame, etc. These emotions arise mostly from critical and negative assessments and expectations of the learning situation and possible outcomes. When learners experience a lack of control over learning outcomes, and at the same time regard the reason for learning as important, they are likely to be affected by increased levels of unpleasant achievement emotions (Putwain et al., 2021). These unpleasant emotions reduce the motivation to approach a learning goal and are related to avoidance behavior, reduced effort, and less time spent for learning (Macher et al., 2013). Unpleasant emotions also divert attention from relevant learning activities and increase attention to inappropriate cues. In this case, they take up a portion of the processing capacity that would be needed for task performance (Eysenck et al., 2007; Macher et al., 2013). They also may trigger the use of rigid learning strategies, such as simple rehearsal and algorithmic procedures (Villavicencio and Bernardo, 2013).

Examples for pleasant achievement emotions are joy, satisfaction, pride, etc. Such pleasant emotions indicate a positive appraisal of the importance, i.e., the value, of the learning task and learning outcomes as well as confidence (Villavicencio and Bernardo, 2013). Positive achievement emotions are related to a learner’s sense of control over the learning situation, to self-regulated learning, and increased effort (Tracy and Robins, 2004). Positive achievement emotions such as enjoyment during learning tasks are mostly associated with higher interest and effort in learning, and less irrelevant thinking (Pekrun et al., 2002).

Achievement emotions may arise from the interaction of stable distal factors like learners’ dispositions and self-assessments (Luttenberger et al., 2018b; Rentzios and Karagiannopoulou, 2021) with characteristics of a learning situations (like uncertainty of learning circumstances). In this interaction the learners’ appraisal of the controllability of the learning situation and the value of learning outcomes plays an important role for the development of unpleasant or pleasant achievement emotions (Kamtsios and Karagiannopoulou, 2020).

### Personal Antecedents for Learning and Achievement Emotions as Distal Learning Factors

Motivation as a long-lasting engagement plays a crucial role when it comes to learning behaviors, emotions, as well as persistence in learning (Dickhäuser and Meyer, 2006). It explains the degree to which an individual makes an effort to achieve a particular goal like a good test score. According to self-determination theory (Deci and Ryan, 2008), intrinsic motivation can be described as an experience of competency, autonomy, and joy that manifests itself in sustainable efforts over a longer range of time. It is autonomous in the sense that it is experienced as self-determined (Ryan and Deci, 2000; Van Soom and Donche, 2014). Furthermore, intrinsic motivation is assumed to be related to deep learning and seeking understanding and personal meaning in learning (Rentzios and Karagiannopoulou, 2021). Intrinsic and extrinsic motivation can be ordered along a continuum with extrinsic motivation taking different forms. Extrinsic motivation may be triggered by external rewards (e.g., a high salary, positive feedback by a teacher) but it may also rely on internal sources within the person itself (e.g., when a student regards her/his chosen study subject as important and accepts the necessity of learning even though it is not always experienced as enjoyment). In that case, one speaks of identified motivation, i.e., a person knows that a behavior is beneficial toward one’s development and adopts that behavior as one’s own (Deci and Ryan, 2008). Extrinsic motivation is assumed to be related to surface learning approaches like rote learning and with minimizing effort for learning (Rentzios and Karagiannopoulou, 2021).

An important disposition with an influence on learning and performance is proneness to anxiety (Chamorro-Premuzic and Furnham, 2003; Luttenberger et al., 2018b). It describes temporally stable individual differences in the tendency to perceive stressful situations as dangerous or threatening (Spielberger, 1985; Endler and Kocovski, 2001; Meijer, 2001). In educational settings, individuals may suffer from anxiety in different academic situations, such as taking a test, preparing for an exam, or attending a class. Proneness to anxiety in academic situations has been linked to the application of less efficient cognitive learning strategies and to a lower investment of individual resources such as attention, effort, or time (Hill and Wigfield, 1984; Pintrich and de Groot, 1990; Matthews et al., 2000; Cassady and Johnson, 2002). Proneness to anxiety is often accompanied by low expectations of success and assessments of low controllability of a learning situation (Lazarides and Raufelder, 2021).

Also, a learner’s assessment of oneself can make a difference for learning. The academic self-concept describes a stable assessment of one’s skills and knowledge in a specific academic domain or generally in school or university. It has an influence on achievement beyond that which may be explained by prior achievement. An individual’s self-concept in a specific domain is influenced by prior learning experiences and, therefore, is also indicative of skills in a specific knowledge domain (Macher et al., 2013; Wimmer et al., 2019). A high (and confident) self-concept is related to feelings of control over learning outcomes (Lazarides and Raufelder, 2021). Additionally, self-concept may be defined according to different frames of reference (Dickhäuser et al., 2002; Ertl et al., 2017; Wimmer et al., 2019). The assessment of one’s academic abilities can be compared to the abilities of peers like co-students, or to an external criterion, like the demands of an academic subject, or it can be a global assessment of one’s academic abilities overall (Luttenberger et al., 2019).

Personal dispositions and stable self-assessments like the academic self-concept, proneness to anxiety, or motivation form important facets of a learner’s identity and act as antecedents and distal factors for learning (Macher et al., 2013).

### Relationships Between Variables and Research Questions

**FIGURE 1:**
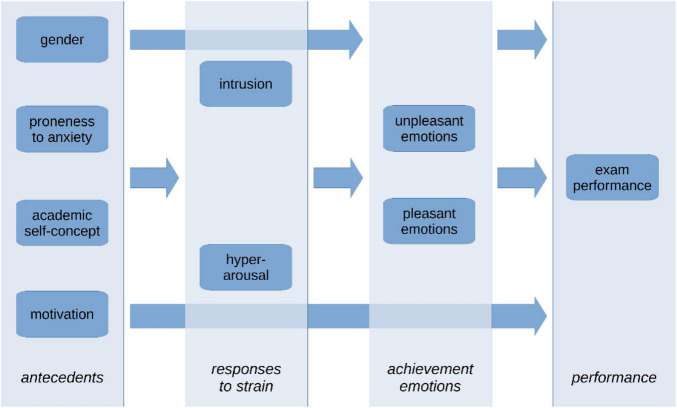
Model for the investigation.

Distal factors for learning as they were explained above constitute relatively stable characteristics that develop through learning experiences from childhood on (Marsh and Martin, 2011; Guil et al., 2019) and that are correlated to each other. For example, higher proneness to anxiety is mostly related to a lower academic self-concept (Macher et al., 2013) as well as to lower intrinsic motivation and to evaluations of learning situations as being uncontrollable (Lazarides and Raufelder, 2021). Research also suggests gender differences in the sense that women are more prone to anxiety and hold a more critical self-concept (Luttenberger et al., 2018a,b) but often they outweigh these adverse self-assessments and dispositions by higher effort and motivation (Macher et al., 2013). The personal characteristics described here constitute antecedents for learning and form the basis of the model to be investigated (see Figure 1). They may function as a buffer or as an amplifier for the evaluation and emotional responses to environmental-related strain as experienced in the COVID-19 pandemics (Dinu et al., 2021; Godoy et al., 2021; Holt-Gosselin et al., 2021).

Therefore, emotional responses to the pandemic situation such as hyperarousal and intrusion were included into the present research. It can be assumed that emotional reactions to the hitherto unknown circumstances of a pandemic also impact students’ learning emotions and experiences in situations like exam preparation (Andrews and Wilding, 2004; Barch et al., 2019).

Pleasant and unpleasant achievement emotions and their impact for learning outcomes are another focus of the study. Unpleasant emotions have been identified as distractors for learning and as triggers of maladaptive learning strategies (Villavicencio and Bernardo, 2013), whereas positive achievement emotions are mostly associated with better learning outcomes (Pekrun et al., 2002). However, it should be considered that achievement emotions are at least to some degree directly related to distal stable factors of learning that influence how learners assess the controllability of a learning situation, how much value they attach to it, or also how much effort they exert, and which learning strategies they prefer (Macher et al., 2012, 2013).

Distal factors of learning may also directly affect learning outcomes as they are reflections of knowledge and skills (e.g., academic self-concept, Wimmer et al., 2019) and/or as they may unfold their effects directly in a test situation (e.g., anxiety, motivation, Macher et al., 2012, 2013).

In the light of these relationships between variables a structural equation model was investigated in order to answer the following research questions on learning in the COVID-19 pandemic: to which degree are achievement emotions either positively or negatively related to exam performance? Which role obtain reactions like intrusive thoughts or hyperarousal in the interplay with achievement emotions and performance? To which degree may distal factors like the academic self-concept, motivation, gender or proneness to anxiety support or impair learning and learning outcomes measured as academic performance in an exam situation?

## Materials and Methods

### Participants

Participants were 314 students at an Austrian university; of these 228 were students in the psychology bachelor’s or master’s degree and 74 were pre-service teachers with Psychology as teaching subject. All participants attended an exam in one of three lectures on Educational Psychology. The sample included 236 female (75.16%) and 78 male students (24.84). The gender composition of the sample corresponds to the gender distribution of psychology students in Austria. Age of the participants ranged from 19 to 45 years (*M* = 24.17, *SD* = 3.54).

### Measures and Procedure

*Intrusion and hyperarousal as reactions to stressful situations* were measured by the German version of the Impact of Event Scale—revised version (Maercker and Schützwohl, 1998). The questionnaire consists of 22 statements that have to be rated on a 4-point Likert scale ranging from “not at all” (1) to “often” (4). Some items were slightly adapted to fit the COVID-19 situation of summer 2020 (e.g., wording of “other things kept reminding me of it,” it was then replaced by “the COVID-19 situation,” while other items could be kept close to the original (e.g., “I had difficulties sleeping through the night”). After removing two items because of their extreme probabilities (*p*_i_ < 0.15) the remaining items were fed into a principal component analysis. Six further items were removed because of substantial side loadings. Items that were not included in the factor solution mostly indicated severe post-traumatic stress or did not fit well to the circumstances a longer-lasting and still ongoing stressful situation. The resulting two factors intrusion (6 items, 2 of which originally belonged to a different factor) and hyperarousal (4 items) showed selectivities of 0.462 ≤ *r*_it_ ≤ 0.695. For intrusion factor loadings lay between 0.429 to 0.858 (with four items above 0.740); internal consistency was 0.804. For hyperarousal factor loadings items lay between 0.612 and 0.695; internal consistency was 0.828.

*Achievement emotions* were measured by the Achievement Emotions Questionnaire (AEQ) (Pekrun et al., 2005). The AEQ consists of three parts which assess achievement emotions (enjoyment, hope, pride, anger, anxiety, shame, hopelessness, and boredom) experienced in the classroom, in test-related situations, and in learning situations like studying at home, e.g., for exam preparation. Only the items on emotions in learning situations were of interest for the present study. The questionnaire consisted of 75 statements that were answered on a five-point Likert scale ranging from “did apply very seldom” (1) to “did apply very often” (5). Students had to assess how the statements described their achievement emotions experienced 1 week prior to the exam. Item examples include “I worry whether I’m able to cope with all my work” (anxiety), “I get angry while studying” (anger), “I enjoy acquiring new knowledge” (enjoyment), “I feel confident when studying” (learning-related hope). Principal component analysis found three factors: pleasant emotions (37 items), unpleasant emotions (18 items), and boredom (12 items). Eight items were removed due to substantial side loadings. Item analysis showed probabilities of 0.153 ≤ *p_*i*_* ≤ 0.793, selectivities of 0.486 ≤ *r*_*it*_ ≤ 0.790, and internal consistencies of 0.928 ≤ α ≤ 0.966. For the present investigation, the factors pleasant and unpleasant emotions were used.

*Proneness to anxiety and motivation* in academic contexts were measured by the LASSI (Learning and Study Strategies Inventory) (Weinstein and Palmer, 2002; Khalil et al., 2020). The LASSI consists of 10 subscales with a total of 80 items (statements) that have to be rated on a 5-point Likert scale from “not at all typical for me” (1) to “very much typical for me” (5). Only the subscales proneness to anxiety in academic situations and motivation were of interest for the present study. Some items had to be slightly adapted to fit the terminology of the university system in the German-speaking countries. Based on the analysis of item probability (0.245 ≤ *p_*i*_* ≤ 0.790), principal component analysis (with promax rotation), item selectivity (0.480 ≤ *r*_*it*_ ≤ 0.767), and Cronbach’s α, eight items were attributed to the subscale proneness to anxiety (α = 0.891) and seven items to the subscale motivation (α = 0.819). Example items for the subscale anxiety are “Even when I am well prepared for a test, I feel very anxious.” The subscale motivation consists of seven items that measure intrinsic and identified motivation (item examples are “Even when learning materials are dull and uninteresting I can manage to keep working until I finish” or “Even if I do not like an assignment, I am able to get myself to work hard on it”).

*Academic self-concept* was measured by three key items from three subscales of the SASK (Scales Academic Self-Concept) (Dickhäuser et al., 2002) which measure an external frame of reference. Students assessed their study-related skills compared to the requirements and demands of their study subject and compared to fellow students on a 7-point Likert scale ranging from low (1) to high (7), and gave an assessment of their study abilities (also on a 7-point scale). Item analysis showed items probabilities of 0.607 ≤ *p_*i*_* ≤ 0.674, item selectivities of 0.640 ≤ *r*_*it*_ ≤ 0.693, and an internal consistency of α = 0.818.

*Academic performance* was measured as examination performance by the number of points achieved. Measurement of points achieved were chosen over grades as they are a more accurate measurement on the level of interval scales. Learning objectives of the exam tasks mainly comprised understanding and application of psychological theories and procedures (see learning taxonomy by Anderson et al., 2001). All participants had taken part in one of three lectures on Educational Psychology. For each course, students could take the exam in either June or July 2020. All exams were structured similarly with a length of 45 min exam time and between 22 and 23 multiple-choice or single-choice tasks and two questions with open answers. Exam points achieved by each participant were transformed to *z*-scores for each exam group.

Furthermore, demographic variables like students’ gender, age, or study subject were recorded.

All participants filled in the questionnaire 3–1 day before attending their exam. The study was performed in accordance with the 1964 Declaration of Helsinki and the American Psychological Association’s Ethics Code. The Ethics Committee of the University of Graz had approved the study. Participation in the research was voluntary. All participants gave written consent to participate in the study and to confirm that their data were used in an empirical study. Statistical analyses were carried out with R 4.1.1 and the lavaan 0.6.9 package for structural equation modeling (Rosseel, 2012).

## Results

Descriptive statistics and correlations between the investigated variables are presented in Tables 1, 2.

**TABLE 1:** Descriptive statistics of the variables of interest in the whole sample and for the subsamples of female and male students.

	*M*	*SD*	*Mdn*	Min	Max
*Age*	24.17	3.54	23.00	19.00	45.00
Women	23.84	3.39	23.00	19.00	45.00
Men	25.18	3.80	24.00	19.00	35.00
*Academic self-concept*	4.83	0.94	5.00	1.00	7.00
Women	4.82	0.87	5.00	2.00	7.00
Men	4.88	1.11	5.00	1.00	7.00
*Intrusion*	2.05	0.69	2.00	1.00	3.83
Women	2.10	0.71	2.17	1.00	3.83
Men	1.91	0.63	1.83	1.00	3.17
*Hyperarousal*	1.77	0.76	1.50	1.00	4.00
Women	1.84	0.79	1.75	1.00	4.00
Men	1.53	0.62	1.25	1.00	3.50
*Unpleasant emotions*	2.13	0.80	1.95	1.00	4.60
Women	2.21	0.80	2.10	1.00	4.60
Men	1.88	0.72	1.68	1.00	3.95
*Pleasant emotions*	2.83	0.71	2.83	1.06	4.72
Women	2.82	0.68	2.83	1.33	4.67
Men	2.85	0.78	2.89	1.06	4.72
*Anxiety*	2.48	0.98	2.38	1.00	5.00
Women	2.61	0.97	2.50	1.00	5.00
Men	2.08	0.90	1.88	1.00	4.63
*Motivation*	3.73	0.68	3.86	1.43	5.00
Women	3.80	0.64	3.86	1.43	5.00
Men	3.51	0.74	3.50	1.86	5.00
*Academic performance*	0.07	0.95	0.20	–2.52	1.86
Women	0.11	0.96	0.27	–2.52	1.86
Men	–0.05	0.92	–0.01	–2.26	1.84

**TABLE 2:** Correlations between the investigated variables.

Variables	1	2	3	4	5	6	7	8	9
(1) Gender		0.049	−0.111*	−0.166**	−0.203**	0.012	−0.242**	−0.181**	–0.085
(2) Academic self-concept	0.049		−0.115*	–0.106	−0.396**	0.229**	−0.359**	0.292**	0.177**
(3) Intrusion	−0.111*	−0.115*		0.628**	0.445**	0.035	0.409**	–0.001	–0.048
(4) Hyper-arousal	−0.166**	–0.106	0.628**		0.521**	–0.097	0.462**	–0.104	–0.069
(5) Unpleasant emotions	−0.203**	−0.396**	0.445**	0.521**		−0.232**	0.664**	−0.232**	−0.172**
(6) Pleasant emotions	0.012	0.229**	0.035	–0.097	−0.232**		–0.015	0.360**	0.014
(7) Proneness to anxiety	−0.242**	−0.359**	0.409**	0.462**	0.664**	–0.015		–0.099	–0.087
(8) Motivation	−0.181**	0.292**	–0.001	–0.104	−0.232**	0.360**	–0.099		0.233**
(9) Academic performance	–0.085	0.177**	–0.048	–0.069	−0.172**	0.014	–0.087	0.233**	

*Pearson and Spearman, respectively.*

*p < 0.05, **p < 0.01.

**FIGURE 2:**
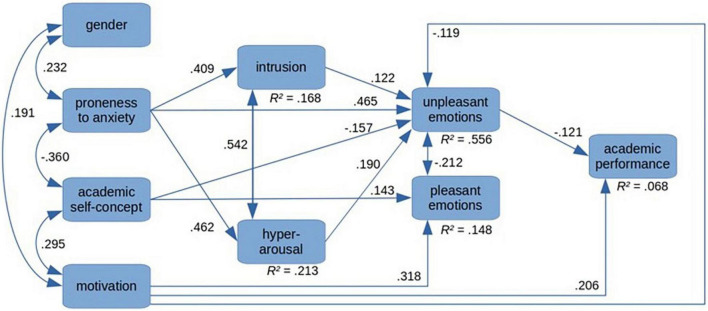
Structural equation model (SEM).

Structural equation modeling was used to investigate the relations between the variables of interest. After eliminating all non-significant paths, the model shown in Figure 2 shows all significant paths with their corresponding values. The χ^2^ test and descriptive fit indices suggest a good model fit (χ^2^[11] = 17.957, *p* = 0.083, χ^2^/*df* = 1.632, CFI = 0.988, SRMR = 0.026, RMSEA = 0.045). Generally, values of χ^2^/*df* < 2, CFI > 0.95, RMSEA < 0.05, and SRMR < 0.05 are considered as indicators of good model fit (Kaplan, 2009).

### Variables in Direct Relationship With Academic Performance

Only two factors, motivation and unpleasant emotions, had a direct relationship to academic performance. Motivation contributed positively (γ = 0.206) to performance. The mean in the sample is *M* = 3.73 (*SD* = 0.68), which is above the scale mean of 2.5 (scale ranging from 1 = low to 5 = high), and it indicates positive student assessments of their motivation level. Unpleasant achievement emotions contributed negatively (γ = −0.121) but with a lower weight than motivation.

### Variables in Direct Relationship With Achievement Emotions

Several variables contributed positively to unpleasant emotions: general proneness to anxiety in academic situations (γ = 0.465), hyperarousal (γ = 0.190), intrusive thoughts (γ = 0.122). For academic self-concept (γ = −0.157) and motivation (γ = −0.119), high values corresponded to a low level of unpleasant emotions. Furthermore, there was a negative relationship between unpleasant and pleasant achievement emotions (γ = −0.212). Academic self-concept (γ = 0.143) and motivation (γ = 0.318) contributed positively to pleasant emotions. For unpleasant emotions, the mean in the overall sample was 2.13 (*SD* = 0.80) and slightly below the scale mean. For pleasant achievement emotions, the mean value of 2.83 (*SD* = 0.71) was slightly above the scale mean of 2.5 and indicates a medium level of pleasant emotions.

### Distal Factors as Antecedents for Learning

Proneness to anxiety contributed to intrusive thoughts (γ = 0.409) and to hyperarousal (γ = 0.462) and to unpleasant emotions (γ = 0.465). Academic self-concept was negatively related to unpleasant emotions (γ = −0.157) and to anxiety proneness (γ = −0.360). It was positively related to pleasant emotions (γ = 0.143) and to motivation (γ = 0.295). With a mean of 4.83 (*SD* = 0.94) for the academic self-concept, it was slightly above the scale mean of 4 (scale from 1 to 7). *Via* motivation academic self-concept was indirectly and positively related to performance.

Gender was neither related to achievement emotions nor to intrusive thoughts, hyperarousal, or performance. It, however, was related to anxiety proneness (γ = 0.232) and to motivation (γ = 0.191) with women showing higher anxiety and higher motivation levels than males.

## Discussion

### Contributions to Achievement Emotions and Performance

According to the SEM, the investigated variables can be distinguished into two groups, variables that contribute negative to performance and variables with a positive contribution.

#### Variables With a Negative Impact on Performance

The SEM suggests that intrusion and hyperarousal experienced in the COVID-19 pandemic and unpleasant emotions “belong together” in the sense that they inhibit academic examination performance directly or indirectly. It is not surprising that dysfunctional cognitions like worries or unwanted thinking about the COVID-19 situation, troubles in concentrating, irritability, etc. were related to unpleasant emotions while learning for the exam. Intrusion and hyperarousal form an immediate threat to effective learning behaviors, such as concentration on the task at hand or use of adequate learning strategies. Such dysfunctional cognitions are also typical expressions of unpleasant achievement emotions. They are usually accompanied by the learners’ impressions of a lack of control over their learning processes (Pekrun et al., 2002; Pekrun et al., 2007). According to control-value theory such effects are even stronger if the outcomes are of high value and importance for a learner (Pekrun et al., 2007). Altogether, one may assume that students with these experiences were less able to build up an adequate knowledge base and were less well-prepared for the exam.

The SEM also points to the importance of the stable distal factors. Proneness to anxiety in academic situations was an important variable for unpleasant emotions and lower performance. Its effects can be explained by control-value theory (Pekrun et al., 2007). Students who generally assume low control over their learning outcomes are prone to a variety of adverse learning behaviors: they invest less effort and time for learning or organize their learning environment less efficiently (Macher et al., 2012). Therefore, it is not surprising that these students also reported of higher levels of mental, emotional, and physical disturbances due to the COVID-19 situation. Proneness to anxiety contributed not only *via* intrusion and hyperarousal to emotions but also had a direct relationship with unpleasant achievement emotions.

#### Variables With a Positive Impact on Performance

Motivation was the only variable in the SEM with a direct and positive relationship with performance. Motivation was measured mainly as intrinsic and identified motivation. A positive motivation level signifies that a certain value is attached to academic goals like passing a course or an examination (Lazarides and Raufelder, 2021). Motivation was also significantly and positively related to the academic self-concept. Usually, an individual’s academic self-concept correlates positively and significantly with former academic experiences and successes (Macher et al., 2013, 2015). Therefore, in accordance to control-value theory, expectancy of success by students with a positive self-concept and a positive assessment of learning activities and learning goals should result in positive learning behaviors and effort and higher learning outcomes (Macher et al., 2015; Luttenberger et al., 2019). Consistent to the theory, motivation and self-concept were negatively related to unpleasant emotions in learning and exam preparation.

Both academic self-concept and motivation increased pleasant emotional experiences in learning and exam preparation. However, contrary to some assumptions, pleasant achievement emotions and academic examination performance (Pekrun et al., 2002; Tracy and Robins, 2004) were not related to better performance. A reason for this might lie in the measurement of achievement emotions that combined different emotions in one factor. Generally, positive emotions can be differentiated according to their level of arousal; an example for a high-arousal emotion would be excitedness, one for lower arousal pleasantness. High arousal can be dysfunctional for learning if it distracts from the task at hand and divert concentration (Kochanska et al., 2000; Sallquist et al., 2009), and this is a possible explanation why in the present study pleasant emotions were not immediately related to academic examination performance.

Similar to other studies, women were more prone to anxiety in academic situations, but showed also showed higher motivation (Macher et al., 2012; Putwain and Daly, 2014; Núñez-Peña et al., 2016).

### Degrees of Strain While Learning in the COVID-19 Summer Semester 2020

The study was carried out at the onset of the COVID-19 pandemic, the first semester in which students experienced extensive restrictions in and insecurities for learning. The sudden change to online learning presented students with new tasks and challenges. To assess strain experienced in this situation the scales intrusion and hyperarousal of the Impact of Event Scale (Maercker and Schützwohl, 1998) had been included. The SEM shows that both, intrusion and hyperarousal, had an effect on students’ experiences of unpleasant emotions in the exam preparation. However, the mean values of students’ hyperarousal and intrusion suggests that strain due to COVID-19 was at a moderate level. There are various reasons for this, either that student did not feel so much encumbered by the situation, e.g., due to the possibility to take an exam and thus being able to progress in their studies. It will not be possible to determine the exact degree to which students suffered from the situation. However, the results are nevertheless important as they point at differentiations between students, i.e., that students with a predisposition of anxiety proneness were much more likely to suffer from hyperarousal and intrusion. Thus, they advise to take tailored measures of support for this group in case of adverse learning conditions.

### Implications for Learning and Instruction

The results give recommendations how learning can be supported by either students themselves or by educators. Such support may not only enhance academic performance but also make learning itself more enjoyable and efficient.

### Alleviating the Impact of Dysfunctional Factors for Learning and Strengthening Favorable Ones

In the study, proneness to anxiety was related to dysfunctional cognitions, unpleasant emotions, and lower performance. While mild anxiety, especially when paired with hope for success, may have a positive influence on academic outcomes by increasing efficiency and intellectual functioning, high levels may result in maladaptive behaviors (Guil et al., 2019). Anxiety unfolds its detrimental effects especially when students value a specific academic goal while at the same time they feel unsure whether they have control over goal achievement (Macher et al., 2015). Measures to alleviate anxiety and thus making learning more pleasant should, therefore, aim to strengthen control over learning outcomes.

Such measure could concern the use of cognitive or meta-cognitive learning strategies as well as resource strategies. For example, time management and prevention of procrastination have been shown to reduce anxiety as well as to support higher academic performance (Macher et al., 2012, 2013, 2015; Luttenberger et al., 2018b). The results of the present study also suggest that anxiety can be reduced by the development of a positive, yet realistic self-concept—all while keeping in mind that improvements in students’ self-concept will be short-lived without enhancing knowledge acquisition and improving achievement (Luttenberger et al., 2019). Further measures of support concern motivation. Intrinsic as well as identified motivation are strengthened when learners recognize that class material is relevant to their everyday live and/or their future profession.

### Reducing Intrusions and Unpleasant Emotions in the Learning Situation

Students as well as educators can take measures in an immediate learning situation. Again, a key for more pleasant and efficient learning is to enhance controllability over the learning outcomes, e.g., by metacognitive learning strategies and monitoring of one’s learning processes or by time management (Saraff et al., 2020). Another means for reducing unpleasant feelings in a learning situation would be positive reappraisal, which means a change in a situation’s evaluation and its potentially threatening characteristics to more positive attributions (Perchtold et al., 2018). In the long range such measures do not only support learning but strengthen a learner’s positive academic self-concept and motivation and thus contribute to a positive identity as a learner.

## Conclusion

The study is not without limitations. One concerns the cross-sectional design. Due to the correlational nature of the data, there are limitations in drawing causal conclusions for the variables recorded at one point in time. However, in the context in which the study took place, it was not possible to record students’ assessments at several points in time. A further limitation of the study lies in the assessment of traumatic stress experiences, measured by the Impact of Event Scale IES—revised version (Maercker and Schützwohl, 1998). Due to low item difficulties, items had to be excluded from use. Similar results were found by Nia et al. (2021) who also investigated stress experiences due to COVID-19 in a non-clinical sample. The scales employed in the present study still mirror cognitive, behavioral, and emotional responses to a traumatic event, though.

Despite these limitations, the results from the present study provide insight learning in the COVID-19 pandemic. For further understandings of students’ handling of adverse circumstances it would be desirable to replicate the study in a non-pandemic situation and investigate to which degree, for example, distal factors moderate the relationship between achievement emotions and performance. Furthermore, it would be worth to investigate other types of adverse learning circumstances and lastly to be able to develop tailored support for students in adverse learning circumstances.

## Data Availability Statement

The raw data supporting the conclusions of this article will be made available by the authors, without undue reservation.

## Ethics Statement

The studies involving human participants were reviewed and approved by the Ethics Committee of the University of Graz. The patients/participants provided their written informed consent to participate in this study.

## Author Contributions

MP, HP-L, BE, DM, SM, and IP made a substantial, direct, and intellectual contribution to the work, and approved it for publication. All authors contributed to the article and approved the submitted version.

## Conflict of Interest

The authors declare that the research was conducted in the absence of any commercial or financial relationships that could be construed as a potential conflict of interest.

## Publisher’s Note

All claims expressed in this article are solely those of the authors and do not necessarily represent those of their affiliated organizations, or those of the publisher, the editors and the reviewers. Any product that may be evaluated in this article, or claim that may be made by its manufacturer, is not guaranteed or endorsed by the publisher.
